# Advances in Research of Hydrogel Microneedle-Based Delivery Systems for Disease Treatment

**DOI:** 10.3390/pharmaceutics16121571

**Published:** 2024-12-09

**Authors:** Juan Cao, Bo Wu, Ping Yuan, Yeqi Liu, Cheng Hu

**Affiliations:** 1 School of Fashion and Design Art, Sichuan Normal University, Chengdu 610066, China; j.cao@sicnu.edu.cn; 2 School of Mechanical Engineering, Sichuan University, Chengdu 610065, China; scu_wubo@stu.scu.edu.cn (B.W.); 2022323020011@stu.scu.edu.cn (Y.L.); 3 School of Mechanical Engineering, Chengdu University, Chengdu 610106, China; yuanping@cdu.edu.cn; 4 National Engineering Research Center for Biomaterials, College of Biomedical Engineering, Sichuan University, Chengdu 610065, China

**Keywords:** hydrogel microneedles, drug delivery systems, biocompatibility, controlled drug release, disease treatment

## Abstract

Microneedles (MNs), composed of multiple micron-scale needle-like structures attached to a base, offer a minimally invasive approach for transdermal drug delivery by penetrating the stratum corneum and delivering therapeutic agents directly to the epidermis or dermis. Hydrogel microneedles (HMNs) stand out among various MN types due to their excellent biocompatibility, high drug-loading capacity, and tunable drug-release properties. This review systematically examines the matrix materials and fabrication methods of HMN systems, highlighting advancements in natural and synthetic polymers, and explores their applications in treating conditions such as wound healing, hair loss, cardiovascular diseases, and cancer. Furthermore, the potential of HMNs for disease diagnostics is discussed. The review identifies key challenges, including limited mechanical strength, drug-loading efficiency, and lack of standardization, while proposing strategies to overcome these issues. With the integration of intelligent design and enhanced control over drug dosage and safety, HMNs are poised to revolutionize transdermal drug delivery and expand their applications in personalized medicine.

## 1. Introduction

**Figure 1:**
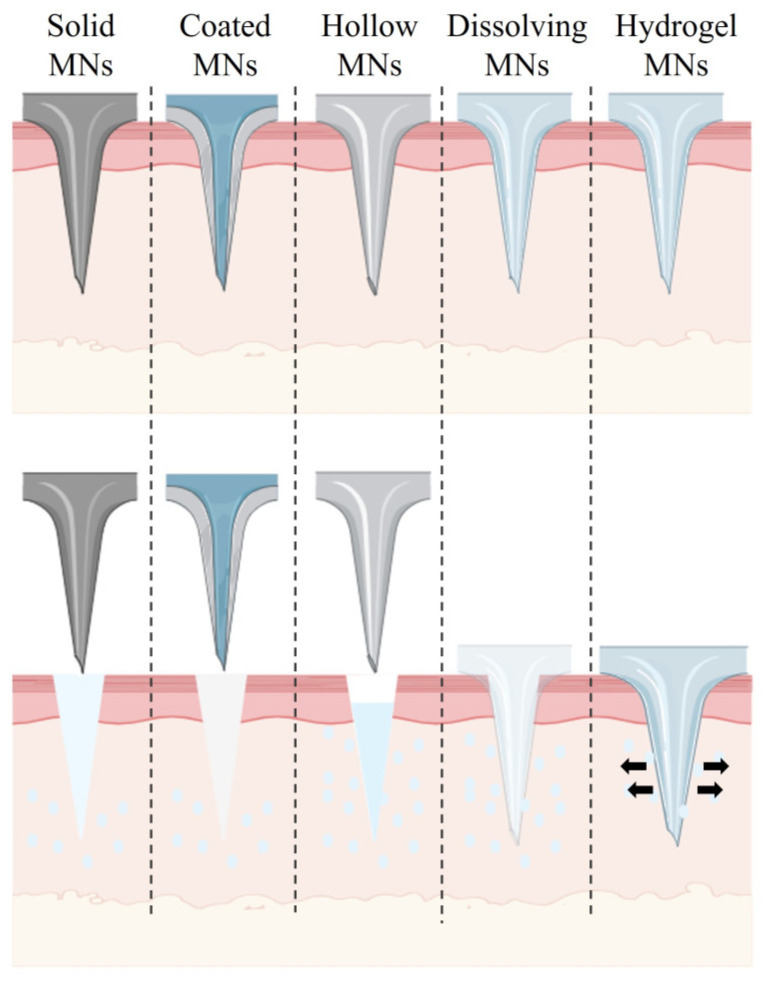
The structure and drug-release mechanisms of these different MN types.

Microneedle (MN)-based delivery systems have emerged as a groundbreaking transdermal drug delivery system (TDDS), offering distinct advantages, including reduced side effects, improved patient compliance, avoidance of first-pass metabolism, and controlled drug release [1,2,3]. Based on the mode of transdermal delivery, MNs can be categorized into five types: solid, coated, hollow, dissolving, and hydrogel MNs [4,5] (Figure 1). The advantages and disadvantages of these different MN types are summarized in Table 1. HMNs are composed of polymer materials cross-linked through chemical or physical processes, granting them unique properties and significant application potential [4]. In their dry state, HMNs possess sufficient mechanical strength to penetrate the skin and absorb interstitial fluid upon insertion, forming continuous and non-blocking microchannels for effective drug delivery. Simultaneously, they maintain mechanical resilience in their hydrated state, enabling the microneedles to be fully removed from the skin without leaving residues [6]. The swelling capacity of HMNs is regulated by the cross-linking density of the polymer’s three-dimensional network structure, which allows for precise control over drug-release rates and loading capacity. Compared to other types of MNs, HMNs feature excellent biocompatibility, high drug-loading capacity, and tunable drug-release rates [7,8]. Recent advances in HMN design, including the development of smart and multifunctional microneedles, have further expanded their potential applications in treating diseases such as cancer, cardiovascular conditions, and chronic wounds.

**Figure 2:**
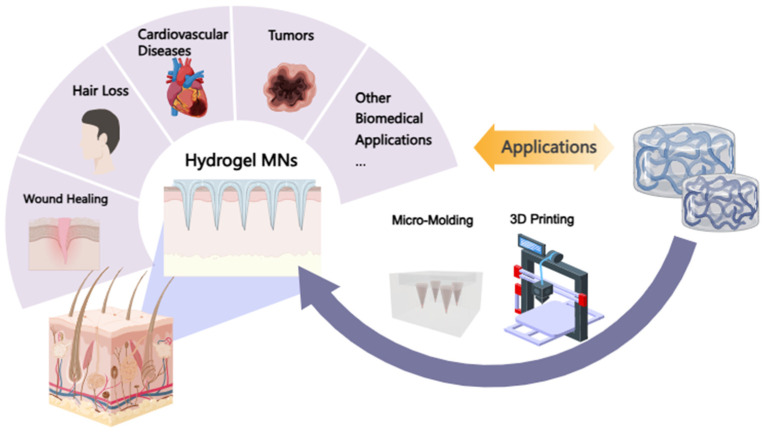
The preparation methods and therapeutic applications of HMNs.

This review provides a comprehensive overview of the current state of HMN systems from a novel perspective, including their different matrix materials, preparation methods, optimized drug-release mechanisms, and therapeutic applications (Figure 2). Additionally, it discusses current limitations related to mechanical strength, drug stability, and clinical standardization of HMNs. Future research on HMNs requires further exploration into material optimization and fabrication techniques to overcome these challenges, thereby promoting their application in the medical field.

## 2. Matrix Materials for HMN

The characteristics of HMNs lie in their water absorption and swelling capabilities, enabling controlled drug-release behavior through the crosslinking density of the hydrogel microneedle matrix material, thereby achieving controlled drug delivery kinetics [23,24]. The release and permeation behaviors of drugs in HMNs are primarily influenced by the properties of the polymers used, with different materials providing unique functionalities. Materials employed in HMNs fabrication are non-toxic, biodegradable, and biocompatible. Commonly used materials, including natural polymers, such as sodium alginate (SA) [25], gelatin [26], hyaluronic acid (HA) [27], collagen [28], and silk fibroin (SF) [29], as well as synthetic polymers like polyvinyl alcohol (PVA) [30] and polymethyl vinyl ether/maleic acid (PMVE/MA) [31], were shown in Table 2.

**Figure 3:**
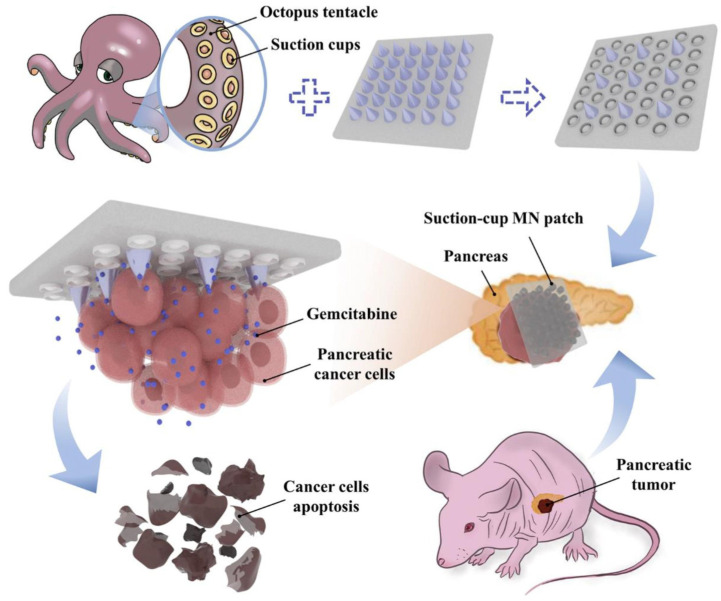
Schematics of the adhesive microneedle patch containing gemcitabine-loaded GelMA for pancreatic cancer treatment. Copyright permission from Fu et al. [33], *Chemical Engineering Journal*, 2022.

Natural polymers typically share characteristics with the human extracellular matrix, promoting cell adhesion and growth, which makes them highly effective in drug delivery. Additionally, using natural polymers is environmentally friendly, as they degrade naturally in the body, reducing environmental impact. Zhou et al. [32] reported a swellable microneedle (SMN) composed of Ca^2+^-cross-linked alginate. Through research on various preparation methods, they successfully developed an alginate-based in situ hydrogel with a planar base, with gelation triggered by EDTA calcium disodium salt, D-(+)-glucono-1, and 5-lactone. In vivo and in vitro results indicated that this SMN exhibited strong mechanical properties and biocompatibility, significantly enhancing transdermal drug delivery. Fu et al. [33] proposed a novel adhesive microneedle patch containing gemcitabine-loaded methacryloyl gelatin (GelMA) for pancreatic cancer treatment. Due to its specific microstructural surface, this HMN patch demonstrated excellent adhesion, allowing it to conform well to the irregular surfaces of tumors (Figure 3). Cheng et al. [35] introduced a cross-linked HA microneedle patch designed to locally deliver PROTACs to treat breast cancer. The microneedle patch can be directly applied to tumor-affected breast tissue. The drug-release curve of the microneedle patch shows that a single drug-loaded patch can release the drug continuously for at least 4 days and penetrate deep into the tumor. A study using an ER^+^ breast cancer mouse model demonstrated that the PROTAC-loaded microneedle patch exhibited excellent efficacy and safety in inhibiting breast tumor growth. Guan et al. [36] developed a novel therapeutic strategy for diabetic skin wounds using a multifunctional silk fibroin methacryloyl hydrogel microneedle patch (MN-PBN-VEGF), encapsulating Prussian blue nanozymes (PBNs) and vascular endothelial growth factor (VEGF) in the tip of the patch and polymyxin in the base layer. Compared to other types of microneedles, this hydrogel microneedle patch demonstrated superior biocompatibility, sustained drug release, pro-angiogenic, antioxidant, and antibacterial properties, offering a new treatment approach for diabetic wounds. Lv et al. [28] used hydrolyzed collagen as the primary matrix and combined it with several commonly used biocompatible materials to develop a flexible base dissolving microneedle (DMN) patch that conforms well to the skin for collagen delivery and skin quality improvement. Niacinamide was further loaded into the microneedles, aiming to achieve dual effects of reversing skin aging and treating skin diseases.

**Figure 4:**
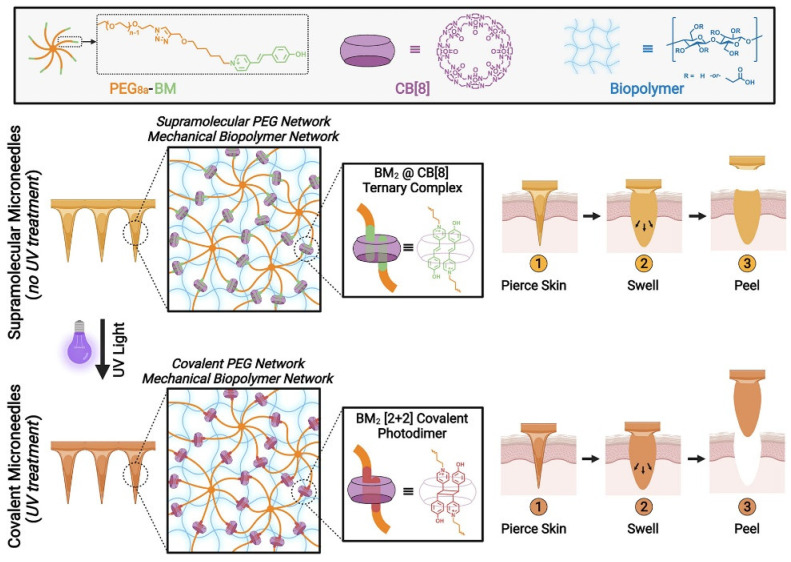
Schematic overview of the design and function of the detachable polymer microneedles. Copyright permission from Ye et al. [39], *ACS Materials Letters*, 2023.

Synthetic polymers, such as polyvinyl alcohol (PVA), polylactic acid (PLA), and polyethylene glycol (PEG), are frequently used to prepare high-performance hydrogel microneedles due to their excellent mechanical properties and tunable physicochemical characteristics. Synthetic polymers allow for precise control over degradation rate, elastic modulus, and mechanical strength through chemical modifications, making them particularly advantageous for applications requiring high precision and stability [46]. Additionally, the surfaces of synthetic polymers can be functionalized to introduce bioactive molecules, enhancing their bioactivity and cell-binding capabilities, thereby improving the drug delivery efficiency of the microneedles [12]. Ye et al. [39] described a strategy for creating detachable polymer microneedles by leveraging supramolecular cross-linking interactions within a PEG network, along with a biopolymer network that provides mechanical reinforcement. This approach enables single-step fabrication of a mechanical network using various biopolymers and allows for the encapsulation of protein payloads for delivery (Figure 4). Zhang et al. [7] proposed an innovative bioinspired adaptive indwelling microneedle with embedded therapeutic exosomes for diabetic wound healing. This microneedle consists of an adjustable PVA hydrogel tip encapsulated with mesenchymal stem cell (MSC) exosomes and a removable 3M medical tape support substrate. In the full-thickness skin wounds of the diabetic rat model, the indwelling microneedle demonstrated the capability to promote tissue regeneration and wound healing, indicating its potential utility and clinical promise for tissue and wound regeneration. Panda et al. [43] used a combination of poly (D, L-lactic-co-glycolic acid) (PLGA) and PVA to optimize the fabrication of microneedles loaded with high-molecular-weight FITC dextran (4 kDa MW). This study demonstrated that incorporating higher molecular weight molecules into PLGA microneedles is an effective strategy for maintaining the release of macromolecules across the stratum corneum/epidermis for transdermal delivery.

Nowadays, it has become increasingly difficult to strictly differentiate between natural and synthetic polymer materials used in modern microneedle manufacturing. Many natural polymers can be chemically modified to alter their properties and applications, such as enhancing mechanical performance, improving biocompatibility, and imparting additional characteristics [2,47]. Similarly, many synthetic polymers can be blended with natural polymers to enhance the overall performance of the polymer material [5,48]. As a result, the polymer materials used in modern hydrogel microneedle fabrication represent a diverse and complex field, requiring consideration of factors such as material source, structure, properties, and applications. In various clinical settings, researchers can control the release properties of polymer materials by adjusting factors like concentration, molecular weight, cross-linking density, and charge characteristics.

**Figure 5:**
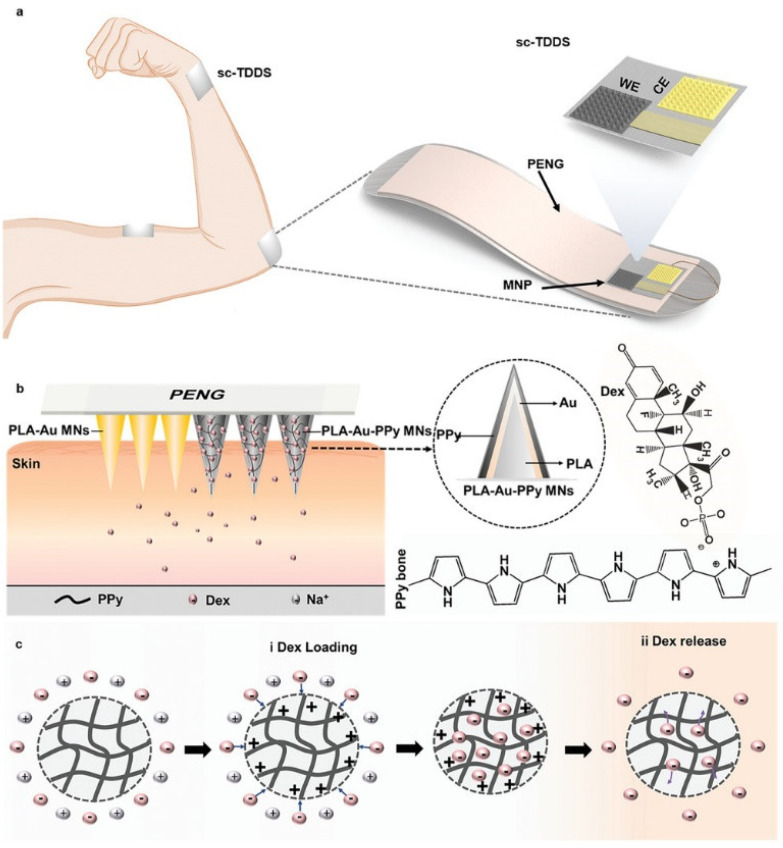
Schematic overview of the design and function of the sc-TDDS. (**a**) The sc-TDDS consists of PENG and MNP; (**b**) MNP includes PLA-Au MNs and PLA-Au-PPy MNs loaded with Dex; (**c**) The mechanism of loading and releasing Dex with PPy. Copyright permission from Yang et al. [45], *Advanced Functional Materials*, 2021.

Additionally, as the requirement grows for the integration, intelligence, and personalization of microneedle-based drug delivery systems, polymers can be blended with different types of substances, such as conductive materials, to create pre-manufacturing solutions for microneedles [49]. Yang et al. [45] designed an automated transdermal drug delivery system (sc-TDDS) with controllable force, featuring two microneedle arrays (Figure 5). In this system, polylactic acid-gold-polypyrrole (PLA-Au-PPy) microneedles loaded with dexamethasone (Dex) serve as the working electrode (WE), while polylactic acid-gold (PLA-Au) microneedles act as the counter electrode (CE). The sc-TDDS controls drug release by harvesting mechanical energy and converting it into electrical energy, releasing 8.5 ng Dex per electric stimulus. Experimental results indicate that this approach significantly outperforms traditional Dex solution coatings, offering a promising method for on-demand transdermal drug release across various therapeutic applications. Lyu et al. [44] developed a novel core-shell microneedle patch for scar-free skin repair. The shell is composed of a GelMA hydrogel loaded with mangiferin, while the core is made of poly(lactic-co-glycolic acid)-dimethacrylate (PGLADMA) loaded with exosomes derived from human mesenchymal stem cells (hMSC). This design leverages the GelMA shell’s rapid anti-inflammatory action during the early stages of wound healing, along with the PGLADMA core’s ability to provide sustained release of hMSC-derived exosomes, promoting inflammation reduction, angiogenesis, and tissue regeneration throughout the healing process. In summary, this microneedle patch not only holds promise for improving wound healing and minimizing scarring but also presents a novel approach for sustained exosome release as a therapeutic strategy.

## 3. Preparation Methods of Hydrogel Microneedles

Currently, commonly used methods for preparing hydrogel microneedles include micro-molding technology and 3D printing. The choice of preparation method typically depends on the type of matrix material and the crosslinking technique employed.

### 3.1. Micro-Molding Technology

**Figure 6:**
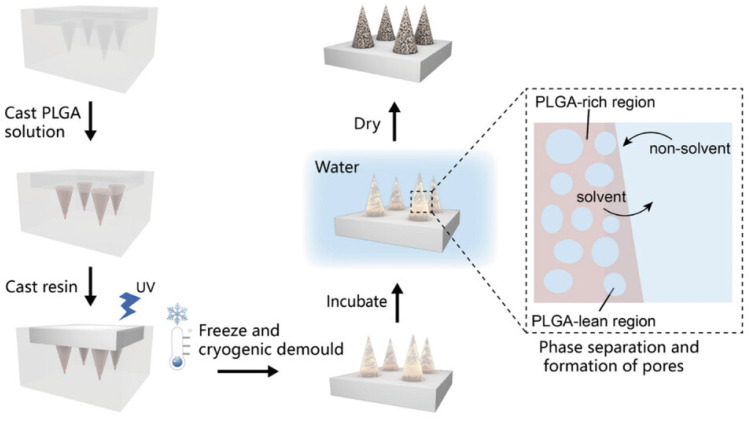
Schematic illustration of the fabrication process of self-healing PMN patch. Copyright permission from Ling et al. [56], *Small*, 2023.

Micro-molding technology is one of the most common methods for producing hydrogel microneedles, offering advantages such as good reproducibility and suitability for large-scale production [50,51]. This method involves fabricating target microneedles on micro-molds, where the mold design must consider factors such as needle density, diameter, and depth to ensure the microneedles meet the requirements for the intended application [52]. After the hydrogel-based microneedle matrix is formed and adapted in the mold, it typically undergoes freeze-drying or other necessary drying treatments to remove excess liquid and residual mold materials [53,54]. Due to the reusable nature of the molds, multiple hydrogel microneedles can be quickly and easily produced, making micro-molding technology advantageous for optimization and mass production [55]. Micro-molds can be used in micro-molding technology through methods such as polymer casting, laser drilling, injection molding, and hot-press molding. Among these, polymer casting is the most common method, which typically involves three main steps: first, creating a master mold made from a solid material; second, pouring polydimethylsiloxane (PDMS) into the master mold to create a negative mold; and third, forming the final structure of the microneedles within the negative mold [38]. This method is widely used due to its capability for large-scale production. Ding et al. [8] applied micro-molding technology to develop a novel hair loss treatment patch, called VR-MNs, which combines HA hydrogel microneedles with polyhydroxy fatty acid ester (PHA) nanoparticles. The patch co-delivers vascular endothelial growth factor (VEGF) and the hair loss drug Ritlecitinib. The combination of HA and PHA nanoparticles not only enhances the mechanical properties and skin penetration efficiency of the microneedles but also enables minimally invasive, painless, and long-lasting controlled release of VEGF and Ritlecitinib. Ling et al. [56] manufactured a biodegradable self-healing porous microneedle (PMN) patch through low-temperature micro-molding and phase separation processes. The PMN patch consists of porous microneedles made from polylactic acid-glycolic acid (PLGA) copolymer and a supporting substrate made from biocompatible resin (Figure 6). PLGA, with its biodegradability and biocompatibility, allows for sustained drug release over weeks to months. Furthermore, PLGA imparts self-healing capabilities to the PMNs, enabling the pore structures to self-repair at the macroscopic scale. The supporting substrate is made of a non-permeable, hydrophobic biocompatible resin, which effectively prevents drug diffusion into the supporting base during drug loading, thus improving delivery efficiency and minimizing drug waste.

### 3.2. 3D Printing Technology

**Figure 7:**
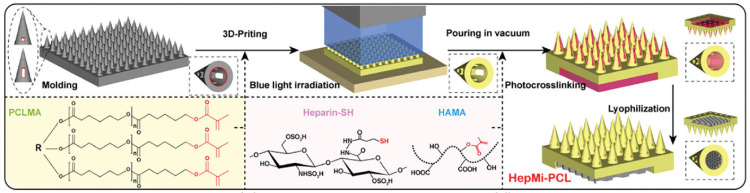
The manufacturing process of HepMi-PCL. Copyright permission from Liu et al. [14], *Advanced Functional Materials*, 2024.

3D printing technology, also known as Additive Manufacturing Technologies (AMT), is a technique that creates physical parts or components by layer-by-layer deposition of adhesive materials (such as powders, liquids, or filaments) based on a three-dimensional digital model designed by a computer [57]. The precision and speed of 3D printing make it an excellent method for preparing hydrogel microneedles [58,59]. By using specific 3D printing techniques such as digital light processing (DLP) and stereolithography (SLA), it is possible to create microneedles with high resolution, stretchability, and a variety of biocompatible materials [37,40]. Compared to traditional mold-based manufacturing methods, 3D printing does not require expensive molds, significantly reducing production costs and improving the efficiency of microneedle production [60]. Additionally, 3D printing offers more flexible design options for length, size, and shape, which can meet diverse and personalized needs in drug delivery, extraction monitoring, and wound healing [61]. Furthermore, hydrogel microneedles made using 3D printing technology also exhibit excellent mechanical properties and adjustability [62]. For example, by adjusting the composition of the hydrogel and the density of metal coordination bonds, microneedles can have an elastic modulus ranging from several kilopascals to several hundred kilopascals, enabling them to match different biological soft tissues [63]. This adjustability provides more possibilities for microneedle design, allowing them to better conform to the biological structures of the human body and improve the delivery efficiency of drugs or biological materials. Shin and Hyun [37] used projection-based light-cured 3D printing technology to create silk fibroin-based hydrogel microneedles. Projection-based light-cured 3D printing is advantageous for directly constructing 3D structures of low-concentration silk fibroin, with the researchers enhancing the printing accuracy by dehydrating the printed hydrogel structure. Liu et al. [14] developed a hollow porous microneedle patch (HepMi-PCL) loaded with smart hydrogels using high-precision bioprinting technology (Figure 7). This patch is designed for rapid indication, smart drug delivery, and minimally invasive treatment of chronic wound infections. To ensure the microneedles have robust mechanical properties, the research team selected polycaprolactone (PCL), a biocompatible material with high mechanical strength and no swelling in water, as the base material and modified it with methacrylation to meet the requirements for light-curing 3D printing. By utilizing self-responsive and therapeutic capabilities, HepMi-PCL demonstrated the potential to accelerate infection diagnosis and treatment, resulting in a more than 200% increase in the healing speed of infected wounds.

## 4. Applications of HMNs in Disease Treatment

HMNs deliver the loaded ingredients directly into the deeper layers of the skin through micro-punctures. The tiny holes formed by the microneedles heal rapidly while avoiding the hepatic first-pass effect and gastrointestinal degradation associated with systemic drug administration. This results in advantages such as strong permeability, ease of administration, and fewer side effects. Consequently, transdermal drug delivery using HMN has been a major research focus in the medical field. Currently, the research on HMN for disease treatment is primarily focused on wound healing, hair loss, diabetes, cardiovascular diseases, and cancer treatment.

### 4.1. Wound Healing

**Figure 8:**
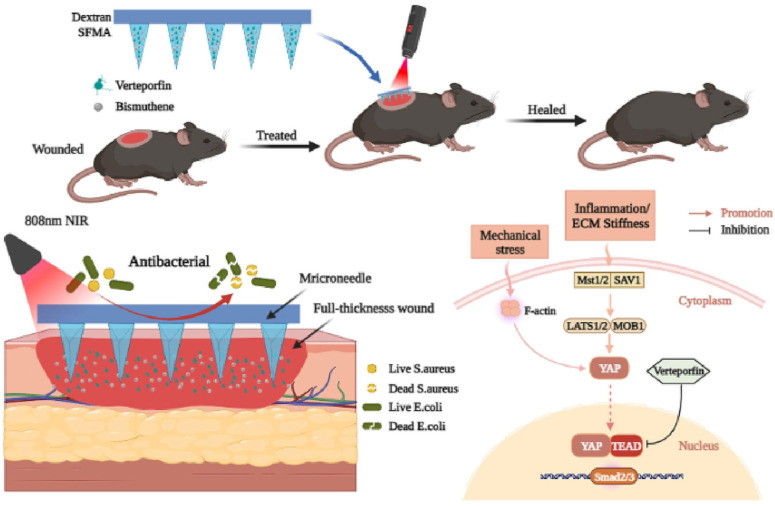
Schematic of microneedle (MN) patch (denoted as Bi/Vp@MN) for scarless wound healing. Copyright permission from Wei et al. [71], *Chemical Engineering Journal*, 2023.

The healing of skin damage caused by severe mechanical trauma or chronic diseases, such as diabetic foot ulcers, is often challenged by issues like tissue damage, microbial infection, intense inflammatory responses, and hypertrophic scar formation [64,65]. In recent years, hydrogel microneedles have been widely applied to promote wound healing. For instance, Yuan et al. [42] developed a sustained-release microneedle patch containing GelMA/PEDDA hydrogel, which maintains the bioactivity of exosomes and drugs in vitro. In addition, the unique physical structure of the microneedle prevents it from being tightly attached to the wound skin. It promotes cell migration and angiogenesis by slowly releasing exos and tazarotene in the deep layer of the skin. The study suggests that GelMA/PEGDA@T^+^exos microneedle patches hold promise as a clinical treatment for diabetic wound healing. In diabetic non-healing wounds, the main obstacles to effective drug penetration are purulent secretions and wound scabs, which microneedle patches can overcome effectively. Tian et al. [66] innovatively integrated taurine into CeO_₂_ nanoparticles and encapsulated them in GelMA hydrogel to create the CTH@MN patch system. This multifunctional system shows remarkable efficacy in eliminating ROS, inhibiting excessive inflammatory responses by macrophages, and restoring a balanced wound microenvironment, offering new strategies and perspectives for treating non-healing diabetic wounds. Wound healing in adult skin often results in scar formation [67]. Transdermal delivery of relevant drugs is considered one of the most convenient and effective strategies, as it can penetrate dense scar tissue and deliver drugs directly to the affected area [68,69,70]. To address scarless healing, Wei et al. [71] developed a biocompatible detachable HMN system. They integrated bismuth (Bi) nanosheets as a photosensitive drug and verteporfin (Vp), an inhibitor of the YAP signaling pathway, into a pyramid-shaped microneedle array. The microneedle system has sufficient mechanical strength to penetrate the skin, and when the separable connection degrades, the tip can remain in the tissue for sustained active release, and the substrate can be safely removed (Figure 8). The experimental results demonstrated that wounds treated with Bi/Vp@MN combined with photothermal stimulation exhibited reduced fibrosis, increased collagen deposition, and improved tissue remodeling between days 30 and 90. These findings provide compelling evidence that the MN platform with Vp inhibitor release can enhance skin wound healing while simultaneously suppressing scar formation in a mouse model. To target pathological scars, Li et al. [72] developed a novel microneedle patch based on the adipose-derived stem cell concentrated conditioned medium (ADSCC-CM) cross-linked keratin hydrogel, used to load triamcinolone (TA). This patch provides synergistic effects from dual-drug delivery of ADSCC-CM and TA to reduce inflammation and modulate myofibroblast behavior. Experimental results show that this hydrogel microneedle patch (TA@AC-MN) can significantly alleviate scar formation by reducing the inflammatory response and enhancing healthy skin regeneration, offering a new self-managing and minimally invasive strategy for treating pathological scars. Several natural herbal medicines show notable potential in promoting wound healing, but challenges remain in optimizing formulations and structures to maximize their efficacy. To address this, Luan et al. [73] proposed a novel microneedle patch for delivering traditional Chinese medicine to treat infected wounds. The microneedles, made from water chestnut starch and aloe gel, exhibit excellent biocompatibility. Following gelatinization and aging, the starch framework provides robust structural support to the microneedles, while the intricate cross-linked matrix facilitates the sustained and gradual release of berberine, ensuring prolonged antibacterial effects throughout the healing process. This study highlights the advantages of combining traditional Chinese medicine with microneedles, opening new avenues for the application of traditional medicine.

### 4.2. Hair Loss

**Figure 9:**
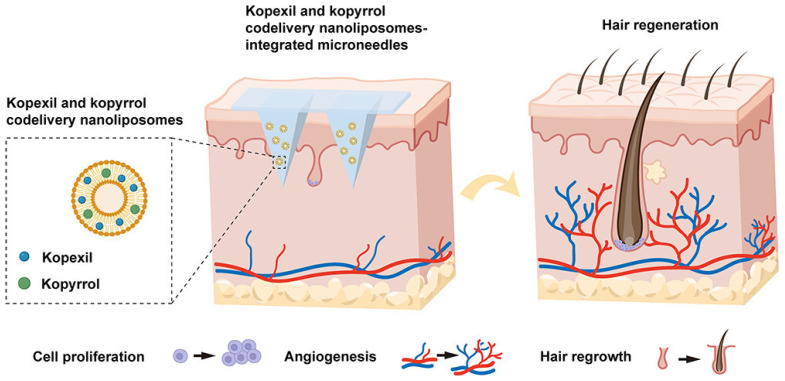
Schematic of a codelivery NLPs-integrated MNs (KK-NLPs@MNs) delivery platform for the treatment of AGA. Copyright permission from Zhang et al. [77], *ACS Applied Materials and Interfaces*, 2024.

In hair loss treatment research, common drug delivery methods include topical application or intradermal injection, as well as the use of roller microneedles or fractional lasers to enhance subcutaneous penetration of topically applied drugs [74]. Due to the limited transdermal permeability of bioactive factors, treatment efficacy tends to be low, while invasive drug delivery can cause discomfort and inconvenience for patients. Hydrogel microneedle technology combines the benefits of traditional intradermal injections with the convenience of transdermal delivery, offering a convenient, minimally invasive, and painless method for hair loss treatment [8,75]. For instance, Yin et al. [76] developed a soluble microneedle patch that incorporates PLGA microspheres for sustained drug release, achieving prolonged delivery of minoxidil for treating androgenetic alopecia. Compared to traditional methods, this approach yielded similar or even enhanced therapeutic effects while reducing the frequency of application, presenting a promising strategy for long-term hair loss treatment. In another study, Zhang et al. [77] encapsulated two key anti-hair loss agents, diaminopyrimidine oxide (kopexil) and pyrrolidinyl diaminopyrimidine oxide (kopyrrol), within nanoliposomes and further incorporated them into dissolvable hyaluronic acid microneedles, creating an innovative microneedle delivery system (KK-NLPs@MNs) for effective treatment of androgenetic alopecia (AGA) (Figure 9). In the AGA mouse model treated with KK-NLPs@MNs and minoxidil, a notable increase in skin thickness was observed. In the KK-NLPs@MNs group, 79.8% of hair follicles (HFs) transitioned into the anagen phase, which was statistically significant compared to the minoxidil group (78.5%, *p* < 0.05). It is worth mentioning that the doses of active ingredients in the KK-NLPs@MNs group (1.5 mg for kopexil and 0.75 mg for kopyrrol) were significantly lower than those in the minoxidil group (6 mg for minoxidil), yet they exhibited comparable effects, thereby demonstrating the superiority of this MNs delivery system. Activating hair follicle stem cells (HFSCs) for follicle regeneration offers hope for hair loss treatments, though designing an efficient, user-friendly method remains challenging. Yang et al. [78] proposed a detachable microneedle patch-mediated drug delivery system, primarily made from keratin derived from hair, for sustained HFSC activator release. Combined with mesenchymal stem cell (MSC)-derived exosomes and small molecule drug UK5099, this microneedle device improved treatment efficacy even at lower doses. Alopecia areata is an autoimmune condition characterized by localized hair loss [79]. Younis et al. [80] developed a novel therapy for treating alopecia areata, designing a hydrogel microneedle patch that can be painlessly applied to the scalp to release immunomodulatory drugs, helping patients rebalance immune responses at the application site and prevent autoimmune attacks on hair follicles. In a mouse experiment, the team observed that this therapy not only induced hair regrowth but also significantly reduced inflammation at the treatment site, without triggering systemic immune responses elsewhere in the body.

### 4.3. Cardiovascular Diseases

**Figure 10:**
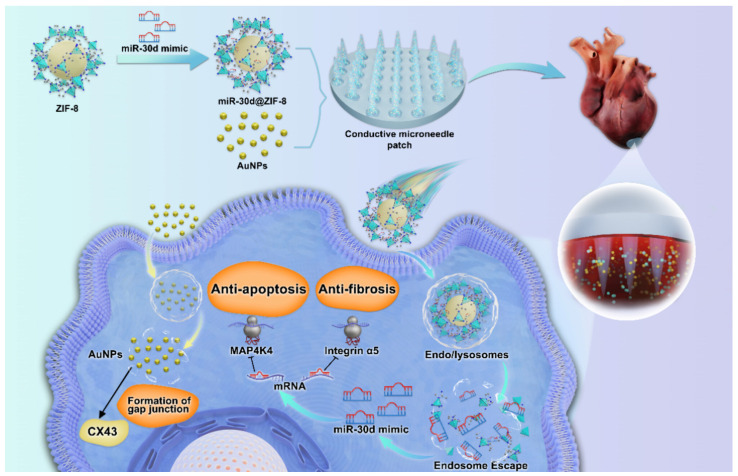
A conductive microneedle patch and miR-30d nano delivery system are integrated to alleviate myocardial ischemia-reperfusion injury (I/RI). Copyright permission from Chen et al. [89], *ACS Nano*. 2024.

Cardiovascular diseases are the leading cause of death worldwide, and microneedles offer a promising new approach to treating these diseases while improving therapeutic outcomes [81,82]. Inhibiting fibroblast activation during the mature phase of cardiac repair can improve cardiac remodeling and function after myocardial infarction (MI) [83,84]. Based on this concept, Chen et al. [85] designed a GelMA hydrogel microneedle patch that loads galunisertib at the microneedle tips to enable targeted myocardial drug delivery. This approach highlights the potential of microneedle patches as an advanced drug delivery platform, capable of locally and minimally invasively delivering antifibrotic agents to prevent myocardial fibrosis, thereby treating myocardial infarction and promoting cardiac repair. In another study, Yuan et al. [86] developed a biocompatible gelatin-based microneedle patch loaded with exosomes containing antifibrotic microRNA-29b (miR-29b) mimics to prevent excessive cardiac fibrosis following myocardial infarction. When applied to infarcted myocardium in a mouse myocardial infarction model, this microneedle patch increased retention of the loaded exosomes within the infarcted tissue, thereby reducing inflammation, limiting infarct size, suppressing fibrosis, and improving cardiac function. Prompt blood reperfusion following myocardial infarction can paradoxically cause ischemia-reperfusion injury (I/RI), a challenge not yet fully addressed by current clinical therapies [87,88]. Chen et al. [89] developed a nanoparticle-microneedle patch system for miR-30d delivery in a mouse model of myocardial I/RI. This system consists of ZIF-8 nanoparticles and a conductive microneedle patch (Figure 10). The ZIF-8 nanoparticles, loaded with miR-30d, use the proton sponge effect to escape endocytosis, thereby avoiding premature degradation in lysosomes. Concurrently, the conductive microneedle patch enables local, efficient, and sustained delivery of miR-30d within the myocardium and releases gold nanoparticles to restore electrical impulses in the infarcted myocardium.

The treatment results showed that compared with the I/RI group, the left ventricular ejection fraction (significantly increased by 16.31 ± 3.50%) and left ventricular fractional shortening (increased by 9.85 ± 2.06%) values of the I/RI + miR-30d + AuNP group had better cardiac performance. These results reveal the therapeutic effects of miR-30d@ZIF-8 nanoparticles and conductive heart patches in improving cardiac function after I/RI. Zhang et al. [90] developed a 3D culture technology that integrates mesenchymal stem cells (MSCs) to generate exosomes (Exo) and used GelMA microneedles as a drug delivery system to enhance the targeting and therapeutic effect of exosomes in brain injury areas. Weak tissue adhesion remains a primary challenge for the clinical translation of microneedle patches [91]. Lu et al. [92] addressed this issue by designing a rigid polymer microneedle with backward-facing barbs, inspired by the structure of a bee stinger, embedded within various elastomeric films to create an interlocking microneedle patch. In myocardial infarction treatment applications, this microneedle patch securely anchors to the beating heart, significantly reducing stress and strain on the infarcted region while maintaining left ventricular function and morphology.

### 4.4. Tumors

**Figure 11:**
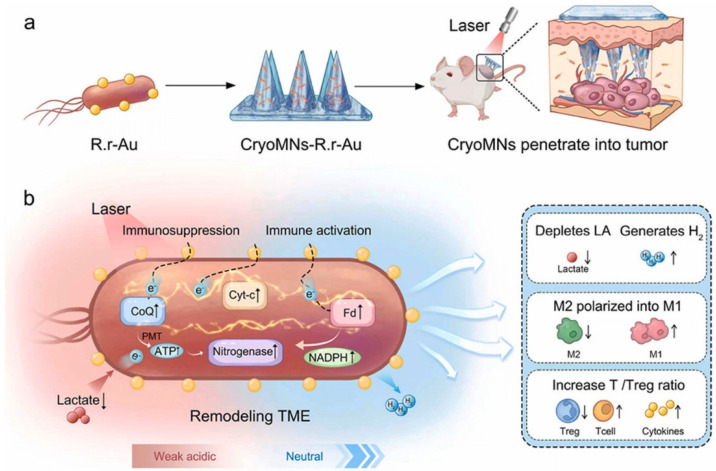
Schematic outline of the fabrication and application of CryoMNs-R.r-Au. (**a**) CryoMNs-R.r-Au utilizes cryomicro needles for transdermal delivery of nanogold-engineered *Rhodospirillum rubrum*, aiming to remodel the tumor microenvironment through optical biotherapy. (**b**) Under laser irradiation, R.r-Au can effectively enhance lactate consumption and hydrogen production via photochemical transformation by transferring electrons into the photosynthetic system of R. *rubrum*, thereby improving antitumor immune activation. Copyright permission from Shi et al. [105], *Bioactive Materials*, 2024.

Cancer is the second leading cause of death worldwide, after cardiovascular disease and traditional treatment methods cause significant harm and suffering to patients [93]. Additionally, patients often need to undergo prolonged oral, injection, or other systemic drug delivery methods, which lead to poor patient compliance and low bioavailability. However, using HMN for transdermal local drug delivery can overcome the skin’s stratum corneum barrier, improve the bioavailability and efficacy of drugs for cancer treatment, and also avoid non-targeted effects associated with systemic exposure [94,95,96]. Local treatment is the preferred approach for treating skin cancer [97,98,99], though it still faces many challenges, such as low delivery efficiency, limited tumor tissue penetration, and suboptimal blood circulation [100]. Shao et al. [101] developed a multilayered, self-heating, multi-stage, therapeutic microneedle patch using a micro-molding method. This patch includes a biodegradable needle tip for sustained drug release, a dissolvable base for rapid drug release, and a functional substrate with a thermal reservoir. In addition to the microneedle-releasing anticancer drugs such as thymoquinone (TQ) and 5-fluorouracil (5-FU) for the treatment of skin cancer, the thermal effect of the microneedles self-heating substrate layer also provides thermal therapy for skin cancer, while simultaneously enhancing drug release through heat. Tumor metabolites are closely related to cancer progression [102], and lactic acid is a characteristic metabolite of the tumor microenvironment (TME), which reflects tumor progression and drives immune suppression [103,104]. Shi et al. [105] proposed an anti-tumor strategy by developing a nanogold-engineered *Rhodospirillum rubrum* (R.r-Au) that consumes lactate and produces hydrogen gas for photobiological therapy. This study used a cryogenic micro-molding method to construct a transdermal therapeutic cryogenic microneedle (CryoMNs) patch integrated with R.r-Au to effectively deliver live bacterial drugs (Figure 11). The results of a 26-day treatment experiment showed that compared to other groups, the group treated with CryoMNs-R.r-Au (^+^) exhibited a significantly higher proportion of immune cells. The percentages of CD8 T cells, CD4 T cells, and NK cells in B16F10 tumor-bearing mice were 17.0%, 22.3%, and 11.2%, respectively. These strongly support the potential of the CryoMNs-R.r-Au patch as a minimally invasive, in situ delivery method for live bacterial drugs for tumor photobiological therapy. After malignant skin tumor resection, wound healing failure, manifested as full-thickness skin defects, large cavities, and incomplete tumor tissue excision, is a major cause of prolonged recovery time, poor prognosis, and high recurrence rates. To address this, Lei et al. [106] developed an HA-based microneedle functionalized with biomineralized melanin nanoparticles to simultaneously perform tumor photothermal therapy (PTT) and promote skin tissue regeneration.

## 5. Conclusions and Perspectives

HMNs represent a transformative approach in TDDS, offering significant advantages such as biocompatibility, precise drug release, high drug-loading capacity, and ease of use. By overcoming the limitations of traditional drug delivery methods, HMNs have demonstrated promising applications in diverse fields, including wound healing, hair loss treatment, cancer therapy, and disease diagnostics. The use of advanced natural and synthetic polymers in HMN fabrication has further enhanced their mechanical properties, adaptability, and therapeutic efficacy. Due to their numerous advantages, hydrogel microneedles are increasingly being researched and applied in the medical field, such as in psoriasis [107,108], ocular diseases [109,110], and arthritis [23]. Additionally, the integration of smart technologies, such as biosensors and responsive materials, has expanded the potential of HMNs for personalized medicine and real-time monitoring [54,111,112,113,114]. For example, Yang et al. [115] constructed a microneedle patch made of an intelligent DNA hydrogel system encapsulated with MeHA for rapid sampling and sensitive detection of miRNA biomarkers in skin ISF. Bao et al. [116] developed an MN patch based on a double-cross-linked hydrogel for simultaneously detecting multiple diabetic biomarkers in vivo and in vitro via a colorimetric reaction. This method allows real-time analysis of multiple biomarkers in ISF within minutes, highlighting the significant potential of this wearable MN biosensor for real-time monitoring of biomarkers associated with chronic diseases or aging.

Despite these advancements, several challenges need to be addressed. For instance, HMNs, due to the limited size of their tips, have a limited drug-loading capacity, making it difficult to meet the required dosages for clinical treatment, particularly for sustained-release microneedles, which limits their clinical application. Additionally, although the design and fabrication techniques of microneedles have been well developed in laboratory settings, the small size of MNs, the high precision required for industrial production, and the need for sterile conditions for mass production of drug-loaded MN patches still pose significant challenges for large-scale manufacturing. Finally, the direct penetration of the skin’s stratum corneum during microneedle application requires strict approval and regulation, and currently, there is a lack of quality standards and policy support for microneedle technologies. Looking forward, future research on HMNs should focus on improving drug-loading capacity by enhancing material formulations and structural designs. Additionally, integrating HMNs with smart devices to achieve synchronized body monitoring and intelligent drug release could improve the precision and effectiveness of treatments. Finally, a comprehensive evaluation system for the quality, efficacy, and safety of MNs should be gradually established to guide the research, development, and production quality control of these products. With continued innovation and research, HMNs are poised to revolutionize transdermal drug delivery and contribute significantly to the development of next-generation therapies across various medical fields.

## Figures and Tables

**Table 1:** Comparison of the advantages and disadvantages of 5 types of MNs.

Types	Advantages	Disadvantages	Ref.
Solid MN	Simple preparation method. Excellent mechanical properties.	Needle breakage may easily occur. It’s difficult to control the drug dosage and the administration time.	[2,3,4,9]
Coated MN	Large drug load. The dosage and administration can be controlled.	Complicated preparation process. Having the possibility of pinhole blockage and flow leakage.	[2,10,11,12,13]
Hollow MN	The dose administered is easy to control. The drug-release rate is fast.	Low drug load. There is a risk of early loss. Part of the coating drug is lost in the stratum corneum.	[2,14,15,16,17]
Dissolving MN	The production and operation methods are simple. The dose is manageable. High biocompatibility.	Poor mechanical properties. High demand for matrix material. Prolonged duration of action.	[18,19,20]
Hydrogel MN	The operation is simple. Large drug load. Controlled release of the drug.	Poor mechanical properties. Poor stability.	[2,5,8,21,22]

**Table 2:** The matrix materials, drug-loaded ingredients, and characteristics of different HMNs.

Matrix Materials	Drug-Loaded Ingredients	Characteristics	Ref.
SA	Ascorbic acid and tranexamic acid	enhanced transdermal permeability	[32]
GelMA	Gemcitabine	outstanding adhesive ability	[33]
GelMA	L-DOPA	improving the utilization rate of the drug	[34]
HA	Estrogen receptor alpha (ERα)-degrading PROTAC—ERD308 and Palbociclib	high rates of local drug retention (87%)	[35]
HA	VEGF, Ritlecitinib	a rapid onset of the anagen phase, improved hair quality, and greater coverage	[8]
SF	Prussian blue nanozymes (PBNs) and VEGF	excellent biocompatibility, drug-sustained release, proangiogenesis, antioxidant, and antibacterial properties	[36]
SF	Riboflavin	dehydration of the printed hydrogel enhances the sharpness of printed features	[37]
SF/ PNIPAM	dexamethasone sodium phosphate (DEX) or classic anticancer drug 5-fluorouracil (5-FU)	wet bonding ability and multiple delivery modes	[38]
hydrolyzed collagen/SA	Nicotinamide	reversing skin aging and treating skin diseases	[28]
PEG	insulin	offering a simple route of fabricating detachable microneedles using polymer formulations with supramolecular bonding	[39]
PEGDA/VP	Acetyl-hexapeptide 3 (AHP-3)	effective wrinkle management	[40]
PVA	mesenchymal stem cell (MSC)-exosomes	promoting tissue regeneration and diabetic wound healing	[7]
PVP	Lidocaíne hydrochloride	sufficient biocompatibility without causing noticeable irritation to the skin	[41]
GelMA/PEGDA	exos and tazarotene	promotes cell migration, and angiogenesis by slowly releasing exos and tazarotene in the deep layer of the skin	[42]
PLGA, PVA, and PVP	FITC Dextran	an effective strategy for the development of a sustained transdermal delivery system for macromolecules	[43]
GelMA/PGLADMA	Mangiferin, human mesenchymal stromal cell (hMSC)-derived exosome	Induce early anti-inflammation, re-epithelization, and vessel formation	[44]
PLA, Au, PPy	dexamethasone (Dex)	on-demand transdermal drug release for various disease treatment	[45]
